# Prevalence, Classification and Factors Associated With Cemental Tears—A Retrospective Observational Cross‐Sectional Study in a Postgraduate Endodontic Clinic

**DOI:** 10.1111/iej.70042

**Published:** 2025-09-30

**Authors:** A. H. C. Lee, A. W. K. Yeung, A. Sigurdsson, M. C. M. Wong, C. F. Zhang

**Affiliations:** ^1^ Faculty of Dentistry The University of Hong Kong Hong Kong SAR; ^2^ New York University College of Dentistry New York USA

**Keywords:** cemental tear, classification, cone beam computerised tomography, periapical radiograph, prevalence

## Abstract

**Introduction:**

There is a lack of epidemiology studies on cemental tears. This study aimed to (i) investigate the prevalence of cemental tears among the patients referred to a Postgraduate Endodontic Clinic, (ii) classify the cases diagnosed with cemental tears, and (iii) assess factors associated with cemental tears.

**Materials and Methods:**

This retrospective, cross‐sectional study included 445 cases/teeth recruited between 1 September 2019 and 31 March 2024 at a University Postgraduate Endodontic Clinic. Information was collated from the clinical records of case history, clinical findings and radiographic interpretations from periapical radiograph (PR) and cone beam computed tomography (CBCT) images. For each case/tooth, cemental tear/s were categorised as either absent or present, and those with cemental tear/s were further classified. Thirty potential factors were studied, including patient‐, jaw‐, tooth‐, previous endodontic treatment and periodontal‐related factors. Factors were individually tested using univariate analysis with Pearson chi‐squared (exact) tests. Any significant factors identified were further subjected to multiple logistic regression analyses based on a forward stepwise regression model (*p* < 0.05).

**Results:**

Cemental tear/s were found in 25 out of 445 cases/teeth, with a prevalence of 5.6%. Sensitivity and specificity of PR in detecting cemental tears were 0.6 and 1.0, respectively. Most cases/teeth detected were classified as Class 2 and 4 (*n* = 19, 76%) or Stage C and D (*n* = 19, 76%). Mean age of the subjects with cemental tears was 58.7 years, with comparable prevalence between males and females. Most cases (72%) with cemental tear/s had root canal treatment initiated or completed. Incisors, increased tooth mobility, severe crestal bone loss and adequate root filling quality were significantly associated with the higher prevalence of cemental tears (*p* < 0.05).

**Conclusion:**

Cemental tears should always be considered as a differential diagnosis in endodontic practice. Clinicians should be particularly vigilant for increased risks of cemental tears in incisors, mobile teeth, teeth exhibiting increased crestal bone loss and those with adequately filled root canals, as they may be misdiagnosed as periodontal‐endodontic lesions. The use of CBCT is crucial for accurate diagnosis, guiding effective treatment planning and improving clinical outcomes in endodontic management.

## 1 Introduction

Cemental tears are characterised by wholly or partially torn cemental or cemento‐dentinal hard tissue fragments from the root surface (Watanabe et al. 2012). These are often misdiagnosed as primary periodontal or endodontic diseases in origin or combined periodontal–endodontic lesions (Lee et al. 2021). The lesions may also mimic vertical root fractures (Jeng et al. 2018; Tai et al. 2007). From the patient's viewpoint, receiving a timely and accurate diagnosis helps mitigate the complications arising from undiagnosed cemental tears, such as pain, persistent swelling or sinus tracts, rapid progression of clinical attachment loss leading to severe periodontal diseases, increased tooth mobility, secondary occlusal trauma, futile treatment attempts and even tooth loss (Lee et al. 2021). On the other hand, from a dental professional's standpoint, the prompt and accurate diagnosis of cemental tears not only relieves the stress, frustration and embarrassment associated with managing patients who continuously report unresolved dental issues and unsuccessful treatments but also safeguards clinicians against potential claims of medical negligence or malpractice from dissatisfied and distressed patients.

Although some factors, including tooth type, gender, age, previous endodontic treatment, history of traumatic injury and heavy occlusal loading, were suggested to be associated with cemental tears, they have yet to be proven (Haney et al. 1992; Harrel 2003; Keskin and Güler 2017; Lin et al. 2011; Tai et al. 2007; Xie et al. 2017). The rarity of cemental tears poses a significant challenge to clinical research on this condition. Only two studies have investigated the prevalence of cemental tears, using either intraoral periapical radiograph (PR) or cone beam computed tomography (CBCT), but not both (Keskin and Güler 2017; Özkan and Özkan 2020). The radiographic presentation of a cemental tear is typically characterised by a radiopaque mass resembling a prickle‐, oblong‐, raindrop‐ or U‐shaped and a flake‐ or calculus‐like appearance surrounded by or within the radiolucent lesion (Chawla and Kumar 2019; Haney et al. 1992; Ishikawa et al. 1996; Müller 1999; Pedercini et al. 2021; Qari et al. 2019; Xie et al. 2017). A thorough evaluation of the two‐dimensional radiographs was considered an essential diagnostic procedure to detect the presence of cemental tear/s (Lin et al. 2011). However, the diagnostic value of two‐dimensional radiographs is compromised by their inability to detect the cemental tear/s on a tooth's labial/buccal and/or palatal/lingual aspects, as well as the potential obscurement of the cemental tear/s by the superimposed anatomic structure (Lin et al. 2011). In contrast, a good quality three‐dimensional imaging modality, such as a high‐resolution, small field‐of‐view (FOV) CBCT scan, should theoretically overcome the issues (Patel et al. 2015). Sliced images in the frontal, sagittal and cross‐sectional planes should better appraise the torn cemental fragment/s and their associated bony lesion of a tooth (Cotton et al. 2007; Patel et al. 2009). Besides, the newly proposed classification system by Lee and co‐workers (2021) requires good quality three‐dimensional radiographic images to assess the pattern and extent of apico‐marginal bone loss (i.e., Class) and the number of root surface/s (i.e., Stage) affected by cemental tear/s and their associated bony lesion (Lee et al. 2021). All these signify the vital role of CBCT in diagnosing cemental tear/s. The objectives of this study were:

- To determine the prevalence of cemental tears utilising high‐resolution, small field‐of‐view CBCT and digital PR in patients referred to a university postgraduate endodontic clinic;
- To categorise the cases identified with cemental tears; and
- To evaluate potential factors associated with the occurrence of cemental tears.

## 2 Materials and Methods

### 2.1 Study Design

This retrospective, observational, cross‐sectional study evaluated patients referred to a University Postgraduate Endodontic Clinic between 1 September 2019 and 31 March 2024. The study protocol was approved by the local Institutional Review Board (IRB reference no. UW 20‐447) and conducted in accordance with the Declaration of Helsinki. Reporting adheres to the PROBE guidelines, and informed consent was obtained from all participants (Data S1).

### 2.2 Sample Population

**FIGURE 1:**
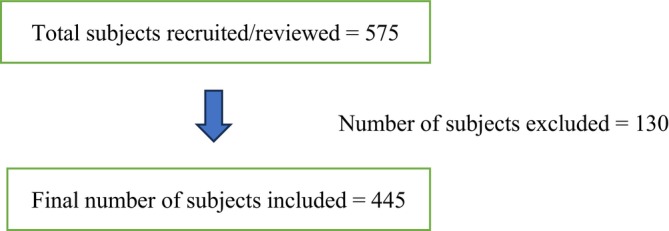
Flowchart illustrating the subjects recruited in this study.

This study reviewed the clinical and radiographical information of 575 patients from various socio‐economic backgrounds who were consecutively referred to the Postgraduate Endodontic Clinic (Figure 1). Recruitment concluded upon reaching the predetermined number of cases that fulfilled the inclusion criteria, that is, 445 patients/cases comprising 445 teeth (Figure 1). The sample size calculation was based on an anticipated study of the prevalence of cemental tears at 5%, as found in the pilot study, rather than the previously reported population prevalence of cemental tears at 2% (Keskin and Güler 2017; Özkan and Özkan 2020), for the 95% confidence level with a precision of 2%. The inclusion criteria were as follows:

– Patients were referred to the Postgraduate Endodontic Clinic between 1 September 2019 and 31 March 2024 for endodontic consultation and management.
– Clinical documentation and records encompassing a comprehensive case history, clinical findings and radiographic interpretations were available.
– Radiographic imaging records encompassing at least one digital PR and good diagnostic quality (with a high resolution and small FOV setting) CBCT scans of the tooth of interest were available.
– Only one randomly chosen tooth was included if a patient had multiple teeth of interest that fulfilled the above criteria.

Exclusion criteria:

- Inadequate or incomplete clinical documentation and records.
- Radiographic imaging records of substandard diagnostic quality.
- Patients who refused or were unable to provide informed consent.

### 2.3 Data Collection

The patient's data was obtained from the clinical and radiographic documentation and records, including the electronic clinical notes, periapical radiographs and reconstructed CBCT imaging. The information collated included: tooth of interest; age; gender; medical history; history of dental trauma; pre‐operative pain; the habit of bruxism; preference for hard diet; incisor and molar relationships; tenderness to percussion; tenderness to palpation; swelling; sinus tract; periodontal probing depth; mobility; the presence of post; type of restoration; the number of proximal contacts; the presence of opposing contacts; pulpal and periapical status; pulp sensibility tests' response (if not previously root‐treated); previous root canal treatment; quality of root filling (adequate or inadequate); the presence, pattern and extent of crestal bone loss; the presence of apico‐marginal bone loss; presence and size of periapical lesion; stage of assessment (i.e., pre‐, intra‐ or post‐operative and review), and the presence of cemental tear/s.

### 2.4 Radiographic Technique and Assessment

Since 2019, a standardised protocol has been in place for obtaining intraoral radiographs and CBCT scans in the Postgraduate Endodontic Clinic with strict supervision. Any PR or CBCT scans not compliant with the protocol, such as those captured at a larger FOV than specified in the inclusion criteria, were excluded from the analysis to ensure the quality of the images studied. The radiographic imaging protocol is described as follows.

The PR was taken by a digital phosphor‐plate sensor aided by a beam‐aiming device (Rinn XCP PSP Fit Phosphor Plate Holder System, Dentsply Sirona) using Heliodent (Sirona, Bensheim, Germany) at the setting of 65 kV and 7 mA; paralleling technique with suitable exposure time was employed depending on the tooth type and size of the patient. The VistaScan intraoral digital system (Durr Dental, Beitigheim‐Bissinger, Germany) was used to scan the exposed sensor to produce the digital PR image. A high‐resolution CBCT scan was taken using Veraview X800 (J Morita Cooperation, Kyoto, Japan) at the setting of 40 mm × 40 mm FOV on ENDO mode with the exposure parameters of 90 kV, 3 mA, 0.08 mm voxel size, 360° rotation and ≤ 17.9 s exposure time, reformatted into 1.5 mm slice thickness. Images were presented on the Apple computer, MacBook Pro (Apple Inc., Cupertino, CA, USA), with a screen pixel resolution of 3024 × 1964. The images were viewed in a quiet room with closed curtains/blinds and dimmed overhead lights. PR imaging was displayed on the Planmeca ROMEXIS viewer (Planmeca OY, Helsinki, Finland), whilst the reconstructed CBCT imaging was displayed on the One Volume Viewer software (J Morita Cooperation, Kyoto, Japan) or Planmeca ROMEXIS viewer software (Planmeca OY). The assessor could enhance the images by manipulating various functions, including magnification, brightness, (reverse) contrast, sharpness, etc. Radiographic signs of a cemental tear were defined as a completely or incompletely detached radiopaque mass described as a prickle‐, chip‐ or flake‐like fragment; calculus‐like spicule; raindrop‐, oblong‐ or U‐shaped structure. The fragment might appear surrounded or within the associated radiolucent bony lesions.

**FIGURE 2:**
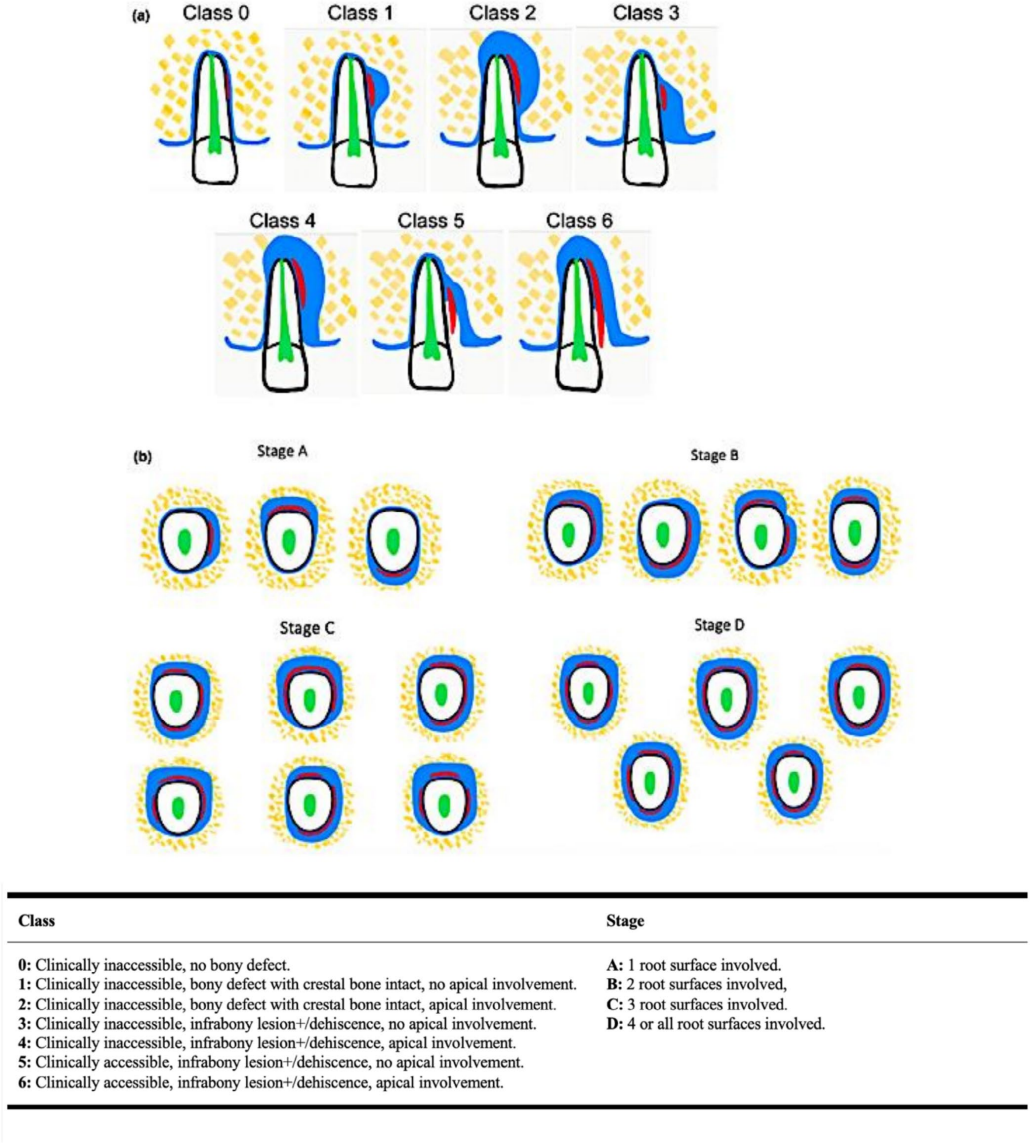
Schematic diagram and summary description of the classification system for cemental tears based on (a) Class and (b) Stage; adapted from Lee et al. (2021).

Cases with cemental tear/s were classified based on the classification proposed by Lee et al. (2021) (Figure 2). The criteria for classifying root filling quality as ‘adequate’ included the presence of a homogenous root filling without any voids or space between the filling and the canal outline, extension 0–2 mm within the radiographic apex/apices, conformity to the canal path without deviation or transportation, as well as the absence of procedural errors (such as separated instruments, root perforations, ledging), gross extrusion and missed or untreated anatomy (Gulabivala and Ng 2023). Any deviations from these criteria resulted in the classification of root filling quality as ‘inadequate’. An endodontist with more than 25 years of clinical experience and a special interest in diagnosing and treating cemental tears was the sole assessor for evaluating the PR and CBCT scans. To assess intra‐observer reliability, one‐tenth of the cases were randomly chosen and re‐evaluated after 3 months.

### 2.5 Statistical Analysis

Statistical analysis was performed using SPSS (IBM SPSS Statistic, Version: 29, IBM Corp., Armonk, NY). The Pearson chi‐squared and Pearson chi‐squared exact tests were used for univariate analysis to identify any significant factors associated with the cemental tear/s. The factors analysed included age, gender, medical history, history of dental trauma, bruxism, hard diet, incisor relationship, molar relationship, tooth type, pre‐operative pain, tenderness to percussion, tenderness to palpation, swelling, sinus tract, periodontal probing depth, mobility, cold test response, EPT response, the number of proximal contacts, the presence of opposing contacts, the presence of post, type of restoration, pulpal and periapical status, root filling quality, root filling extension, root filling extrusion, the presence and severity of crestal bone loss, the presence of apico‐marginal bone loss and periapical lesion size. Subgroupings of each factor are presented in Tables 1 and 2, with the term ‘unspecified’ used to denote missing data. A multiple logistic regression analysis was conducted, including all factors, and backward stepwise selection was used to remove insignificant variables. The level of significance was set at 5%. The diagnostic accuracy of PR was assessed using CBCT as the reference standard. Sensitivity, specificity, positive predictive value (PPV), negative predictive value (NPV) and reliability were computed. Cohen's Kappa Score Statistics was used to evaluate the intra‐observer agreement.

**TABLE 1:** Univariate analysis of factors associated with cemental tears using ^a^the Pearson chi‐squared test and ^b^the Pearson chi‐squared exact test.

Factors	Cemental tear			*p*
	Absent, *n* (%)	Present, *n* (%)	Total, *n* (%)	
	420 (94.4%)	25 (5.6%)	445 (100.0%)	
**Patient‐related**				
Age^a^				
≤ 19	10 (100.0%)	0 (0.0%)	10 (100.0%)	0.128
20–39	110 (98.2%)	2 (1.8%)	112 (100.0%)	
40–59	140 (94.0%)	9 (6.0%)	149 (100.0%)	
≥ 60	160 (92.0%)	14 (8.0%)	174 (100.0%)	
Gender^a^				
Male	139 (92.1%)	12 (7.9%)	151 (100.0%)	0.126
Female	281 (95.6%)	13 (4.4%)	294 (100.0%)	
Medical history^a^				
No	237 (94.8%)	13 (5.2%)	250 (100.0%)	0.600
Yes	172 (93.5%)	12 (6.5%)	184 (100.0%)	
Unspecified	11 (100.0%)	0 (0.0%)	11 (100.0%)	
History of dental trauma^b^				
No	27 (96.4%)	1 (3.6%)	28 (100.0%)	0.073
Yes	41 (87.2%)	6 (12.8%)	47 (100.0%)	
Unspecified	352 (95.1%)	18 (4.9%)	370 (100.0%)	
Bruxism^b^				
No	310 (95.4%)	15 (4.6%)	325 (100.0%)	0.310
Yes	70 (92.1%)	6 (7.9%)	76 (100.0%)	
Unspecified	40 (90.9%)	4 (9.1%)	44 (100.0%)	
Hard diet^b^				
No	299 (94.9%)	16 (5.1%)	315 (100.0%)	0.582
Yes	55 (94.8%)	3 (5.2%)	58 (100.0%)	
Unspecified	66 (91.7%)	6 (8.3%)	72 (100.0%)	
**Jaw‐related**				
Incisor relationship^b^				
Not applicable	12 (92.3%)	1 (7.7%)	13 (100%)	0.896
Class 1	247 (94.3%)	15 (5.7%)	262 (100.0%)	
Class 2	69 (93.2%)	5 (6.8%)	74 (100.0%)	
Class 3	66 (94.3%)	4 (5.7%)	70 (100.0%)	
Openbite	7 (100.0%)	0 (0.0%)	7 (100.0%)	
Unspecified	19 (100.0%)	0 (0.0%)	19 (100.0%)	
Molar relationship^b^				
Not applicable	85 (91.4%)	8 (8.6%)	93 (100.0%)	0.376
Class 1	206 (94.1%)	13 (5.9%)	219 (100.0%)	
Class 2	54 (98.2%)	1 (1.8%)	55 (100.0%)	
Class 3	57 (95.0%)	3 (5.0%)	60 (100.0%)	
Unspecified	18 (100.0%)	0 (0.0%)	18 (100.0%)	
**Tooth‐related**				
Tooth type^b^				
Incisors	88 (86.3%)	14 (13.7%)	102 (100.0%)	< 0.001*
Canines	11 (84.6%)	2 (15.4%)	13 (100.0%)	
Premolars	68 (95.8%)	3 (4.2%)	71 (100.0%)	
Molars	253 (97.7%)	6 (2.3%)	259 (100.0%)	
Pre‐operative pain^a^				
No	163 (94.2%)	10 (5.8%)	173 (100.0%)	0.086
Yes	250 (95.1%)	13 (4.9%)	263 (100.0%)	
Unspecified	7 (77.8%)	2 (22.2%)	9 (100.0%)	
Tenderness to percussion^a^				
Absence	235 (94.4%)	14 (5.6%)	249 (100.0%)	0.176
Present	176 (95.1%)	9 (4.9%)	185 (100.0%)	
Unspecified	9 (81.8%)	2 (18.2%)	11 (100.0%)	
Tenderness to palpation^a^				
Absence	315 (96.0%)	13 (4.0%)	328 (100.0%)	0.006*
Present	88 (91.7%)	8 (8.3%)	96 (100.0%)	
Unspecified	17 (81.0%)	4 (19.0%)	21 (100.0%)	
Swelling^a^				
Absence	301 (96.2%)	12 (3.8%)	313 (100%)	0.037*
Present	95 (89.6%)	11 (10.4%)	106 (100.0%)	
Unspecified	24 (92.3%)	2 (7.7%)	26 (100.0%)	
Sinus tract^a^				
Absence	302 (96.2%)	12 (3.8%)	314 (100.0%)	0.025*
Present	100 (89.3%)	12 (10.7%)	112 (100.0%)	
Unspecified	18 (94.7%)	1 (5.3%)	19 (100.0%)	
Periodontal probing depth^b^				
3 mm or less	213 (96.4%)	8 (3.6%)	221 (100.0%)	< 0.001*
4‐6 mm	119 (98.3%)	2 (1.7%)	121 (100.0%)	
7 mm or greater	66 (83.5%)	13 (16.5%)	79 (100.0%)	
Unspecified	22 (91.7%)	2 (8.3%)	24 (100.0%)	
Mobility^b^				
No	291 (99.0%)	3 (1.0%)	294 (100.0%)	< 0.001*
Grade 1	49 (75.4%)	16 (24.6%)	65 (100.0%)	
Grade 2	6 (85.7%)	1 (14.3%)	7 (100.0%)	
Grade 3	2 (50.0%)	2 (50.0%)	4 (100.0%)	
Unspecified	72 (96.0%)	3 (4.0%)	75 (100.0%)	
Cold test^b^				
Not applicable	274 (93.8%)	18 (6.2%)	292 (100.0%)	0.585
Negative	84 (95.5%)	4 (4.5%)	88 (100.0%)	
Positive	43 (97.7%)	1 (2.3%)	44 (100.0%)	
Unspecified	19 (90.5%)	2 (9.5%)	21 (100.0%)	
EPT^b^				
Not applicable	276 (93.9%)	18 (6.1%)	294 (100.0%)	0.360
Negative	80 (97.6%)	2 (2.4%)	82 (100.0%)	
Positive	64 (92.8%)	5 (7.2%)	69 (100.0%)	
Number of proximal contacts^a^				
0	8 (88.9%)	1 (11.1%)	9 (100.0%)	0.232
1	111 (97.4%)	3 (2.6%)	114 (100.0%)	
2	301 (93.5%)	21 (6.5%)	322 (100.0%)	
Opposing contact^b^				
Absent	13 (92.9%)	1 (7.1%)	14 (100.0%)	1.000
Present	378 (94.5%)	22 (5.5%)	400 (100.0%)	
Unknown	29 (93.5%)	2 (6.5%)	31 (100.0%)	
Post^a^				
Absent	183 (94.3%)	11 (5.7%)	194 (100.0%)	0.149
Present	37 (88.1%)	5 (11.9%)	42 (100.0%)	
Not applicable	200 (95.7%)	9 (4.3%)	209 (100.0%)	
Type of restoration^b^				
Absent	58 (89.2%)	7 (10.8%)	65 (100.0%)	0.185
Direct restoration	211 (95.9%)	9 (4.1%)	220 (100.0%)	
Indirect restoration	143 (94.1%)	9 (5.9%)	152 (100.0%)	
Unknown	8 (100.0%)	0 (0.0%)	8 (100.0%)	
Pulpal status^b^				
Vital	40 (97.6%)	1 (2.4%)	41 (100.0%)	1.000
Non‐vital	91 (93.8%)	6 (6.2%)	97 (100.0%)	
Root canal treatment	284 (94.0%)	18 (6.0%)	302 (100.0%)	
Other	5 (100.0%)	0 (0.0%)	5 (100.0%)	
Periapical status^b^				
Absence of periapical lesion	62 (98.4%)	1 (1.6%)	63 (100.0%)	0.345
Presence of periapical lesion	330 (93.8%)	22 (6.3%)	352 (100.0%)	
Others	28 (93.3%)	2 (6.7%)	30 (100.0%)	
**Treatment‐related**				
Quality of root filling^a^				
Inadequate	157 (98.1%)	3 (1.9%)	160 (100.0%)	< 0.001*
Adequate	64 (83.1%)	13 (16.9%)	77 (100.0%)	
Not applicable	199 (95.7%)	9 (4.3%)	208 (100.0%)	
Extension of root filling^a^				
0–2 mm	210 (95.5%)	10 (4.5%)	220 (100.0%)	0.622
> 2 mm underextended	197 (93.4%)	14 (6.6%)	211 (100.0%)	
Overextended	13 (92.9%)	1 (7.1%)	14 (100%)	
Extrusion of root filling^a^				
Absent	185 (93.0%)	14 (7.0%)	199 (100.0%)	0.496
Present	37 (94.9%)	2 (5.1%)	39 (100.0%)	
Not applicable	198 (95.7%)	9 (4.3%)	207 (100.0%)	
**Periodontal‐related**				
Crestal bone loss^a^				
Mild (30% or less)	298 (97.1%)	9 (2.9%)	305 (100.0%)	< 0.001*
Moderate (40%–60%)	47 (95.9%)	2 (4.1%)	49 (100.0%)	
Severe (70% or above)	75 (84.3%)	14 (15.7%)	91 (100.0%)	
Apicomarginal bone loss^b^				
Absent	379 (96.4%)	14 (3.6%)	412 (100.0%)	< 0.001*
Present	41 (78.8%)	11 (21.2%)	52 (100.0%)	
Periapical lesion size^b^				
≤ 10 mm	390 (94.7%)	22 (5.3%)	143 (100.0%)	0.418
> 10 mm	30 (90.9%)	3 (9.1%)	33 (100.0%)	

* The factor found to be significant with *p* < 0.05.

**TABLE 2:** Multivariate analysis of factors associated with cemental tears using Logistic Regression models.

Factors	OR (95% CI)	*p*
Tooth type		
Incisors^a^		< 0.001*
Canines	6.14 (0.78–48.18)	0.084
Premolars	0.68 (0.13–3.49)	0.647
Molars	0.08 (0.02–0.34)	< 0.001*
Mobility		
No^a^		< 0.001*
Grade 1	26.87 (5.93–121.81)	< 0.001*
Grade 2	18.69 (1.23–283.50)	0.035*
Grade 3	48.27 (2.33–1000.44)	0.012*
Unspecified	3.00 (0.51–17.80)	0.227
Crestal bone loss		
Mild (30% or less)^a^		0.002*
Moderate (40%–60%)	1.33 (0.21–8.45)	0.764
Severe (70% or above)	10.83 (2.85–41.21)	< 0.001*
Quality of root filling		
Inadequate^a^		0.026*
Adequate	8.38 (1.73–40.69)	0.008*
Not applicable (untreated)	3.68 (0.73–18.66)	0.116

^a^ The reference category.

* The factor found to be significant with *p* < 0.05.

## 3 Results

A total of 445 patients/cases comprising 445 teeth were included. Cemental tears were detected in 25 cases, with a prevalence of 5.6%.

Nine factors were found to be significant from univariate analysis (*p* < 0.05) (Table 1), which included tooth type (*p* < 0.001), tenderness to palpation (*p* = 0.006), swelling (*p* = 0.037), sinus tract (*p* = 0.025), periodontal probing depth (*p* < 0.001), mobility (*p* < 0.001), quality of root filling (*p* < 0.001), crestal bone loss (*p* < 0.001) and apico‐marginal bone loss (*p* < 0.001). When all the factors were subjected to multivariable analysis, four factors were found to be significant, including tooth type, mobility, crestal bone loss and quality of root filling (*p* < 0.05) (Table 2).

For tooth type, cemental tears were found to be significantly more prevalent in incisors than in molars (OR 0.08, CI 0.02–0.34, *p* < 0.001) (Table 2). Cemental tears also exhibited significantly increased tooth mobility compared to those without (*p* < 0.001); the odds of cases exhibiting Grade 3 mobility were over 48‐fold more likely to be found in cases with cemental tears (CI 2.33–1000.44, *p* = 0.012), while the odds of those with Grade 1 and Grade 2 mobility were approximately 27‐fold (CI 5.93–121.81, *p* < 0.001) and 19‐fold (CI 1.23–283.50, *p* = 0.035) greater, respectively. For crestal bone loss, cases with cemental tears were significantly associated with severe crestal bone loss compared to those presenting with only mild bone loss (*p* = 0.002); the odds of severe bone loss were almost 11‐fold higher in cases with cemental tears (CI 2.85–41.21, *p* < 0.001) (Table 2). Adequate root filling quality was another significant factor identified (*p =* 0.026), with the odds being more than 8‐fold higher (CI 1.73–40.69, *p* = 0.008) in cases with cemental tears.

The distribution of cemental tear cases according to classifications was: (i) Class 4/Stage C and Class 4/Stage D (*n* = 4 each); (ii) Class 2/Stage C and Class 4/Stage B (*n* = 3 each); (iii) Class 2/Stage B and Class 2/Stage D (*n* = 2 each); and (iv) Class 1/Stage C, Class 2/Stage A, Class 3/Stage C, Class 5/Stage C, Class 5/Stage D, Class 6/Stage C and Class 6/Stage D (*n* = 1 each) (Tables 3 and 4). When ‘Class’ and ‘Stage’ were considered independently, more than 3/4 of the cases with cemental tears were classified as either Class 2 and 4 or Stage C and D (Tables 3 and 4). The Cohen's Kappa Score for the intra‐observer reliability was 1.0, representing excellent agreement among the sole observer in identifying the presence or absence of cemental tears and their classification.

**TABLE 3:** Descriptive statistics of cases with cemental tears.

No	Tooth	Classification		Age/Sex	Stage of assessment	Previous RCT; if no, pulp tests response	Pain	Dental trauma	Bruxism	Hard diet	Pattern and max. crestal bone loss	Swelling/sinus tract; mobility	Periapical lesion (mm)	Probing depth (mm)	Post; restoration	Proximal contacts
1.	16	Class 3	Stage C	67/F	Pre‐operative	Previously treated	No	—	No	No	Intrabony, furcation; 100%	Yes; M3	10 × 6 × 8	8	No; crown	1
2.	21	Class 1	Stage C	62/F	Review	Previously treated	Yes	—	No	—	No; 0%	Yes; M1	1 × 3 × 3 (lateral)	≤ 3	No; temporary crown	2
3.	32	Class 4	Stage D	59/F	Pre‐operative	Previously treated	—	—	—	—	No; 0%	—; —	6 × 6 × 6	—	No; composite	2
4.	36	Class 4	Stage B	63/M	Pre‐operative	Previously initiated therapy	Yes	—	Yes	Yes	Intrabony, furcation; 100%	Yes; M1	2 × 14 × 7	10	n/a; temporary restoration	2
5.	21	Class 5	Stage C	60/M	Pre‐operative	No, +ve	No	No	No	No	Intrabony; 80%	No; M1	0	8	n/a; no	2
6.	21	Class 4	Stage D	44/F	Pre‐operative	Previously treated	Yes	Yes	Yes	No	Intrabony, horizontal; 100%	No; M1	6 × 10 × 10	14	No; composite	2
7.	12	Class 2	Stage D	81/F	Pre‐operative	Previously treated	—	—	—	No	Horizontal; 25%	Yes; M1	6 × 7 × 8	6	Yes; crown	2
8.	46	Class 6	Stage C	61/F	Pre‐operative	Previously treated	Yes	—	Yes	—	Intrabony, furcation; 100%	No; —	10 × 8 × 9	10	No; crown	1
9.	34	Class 4	Stage C	39/F	Review	Previously treated	No	—	Yes	No	Dehiscence; 100%	No; —	4 × 4 × 5	≤ 3	No; composite	2
10.	12	Class 4	Stage C	71/F	Pre‐operative	Previously treated	Yes	Yes	No	No	Intrabony; 100%	Yes; M1	4 × 10 × 7	8	No; composite	2
11.	12	Class 2	Stage A	66/F	Pre‐operative	Previously treated	Yes	—	No	No	Intrabony; 50%	No; M1	3 × 3 × 4 (apical) 4 × 4 × 4 (lateral)	8	No; composite	2
12.	13	Class 2	Stage C	46/F	Pre‐operative	No; +ve	No	—	No	No	No; 0%	Yes; no	7 × 14 × 9	11	n/a; no	2
13.	23	Class 2	Stage C	69/M	Pre‐operative	No; —	Yes	—	No	No	No; 0%	No; M1	7 × 8 × 7	≤ 3	n/a; no	2
14.	34	Class 2	Stage B	33/M	Pre‐operative	No; +ve	No	—	—	No	No; 0%	Yes; —	17 × 9 × 6	≤ 3	n/a; no	2
15.	14	Class 4	Stage C	46/M	Review	Previously treated	No	—	Yes	—	Intrabony; 100%	Yes; M1	4 × 4 × 4	9	No; composite	2
16.	31	Class 4	Stage D	84/M	Pre‐operative	Previously treated	Yes	Yes	No	No	Intrabony; 100%	No; M1	4 × 6 × 4	≤ 3	Yes; crown	2
17.	11	Class 2	Stage C	49/M	Pre‐operative	Previously treated	No	—	No	—	No; 0%	No; M1	6 × 8 × 7	≤ 3	Yes; crown	2
18.	31	Class 2	Stage D	53/M	Pre‐operative	Previously treated	Yes	Yes	No	No	Horizontal; 25%	Yes; M1	7 × 7 × 6	—	No; composite	2
19.	47	Class 6	Stage D	55/F	Pre‐operative	Previously treated	No	—	No	Yes	Intrabony; 100%	No; M2	9 × 9 × 9	10	No; crown	1
20.	32	Class 5	Stage D	71/M	Pre‐operative	No; —	Yes	—	No	No	Intrabony; 50%	No; M1	2 × 1 × 2	≤ 3	n/a; no	0
21.	26	Class 4	Stage D	77/F	Pre‐operative	No; −ve	Yes	—	No	No	Intrabony; 100%	Yes; no	MB 4 × 4 × 6; DB 3 × 5 × 2; P 5 × 8 × 6	14	n/a; no	2
22.	16	Class 4	Stage B	48/M	Pre‐operative	No; −ve	Yes	—	Yes	No	Intrabony, furcation, horizontal; 100%	Yes; M1	B 6 × 5 × 7; P 6 × 10 × 4	9	n/a; no	2
23.	22	Class 4	Stage C	65/F	Pre‐operative	Previously treated	No	—	No	No	Intrabony; 100%	Yes; M3	7 × 10 × 6	11	Yes; temporary crown	2
24.	21	Class 2	Stage B	35/M	Review	Previously treated	Yes	Yes	No	Yes	No; 0%	No; M1	3 × 2 × 3	≤ 3	No; crown	2
25.	31	Class 4	Stage B	66/M	Pre‐operative	Previously initiated therapy	No	Yes	—	—	Intrabony; 100%	Yes; M1	4 × 8 × 5	4	n/a; temporary restoration	2

*Note:* — denotes ‘unspecified’ in the clinical record.

**TABLE 4:** Prevalence of cases with cemental tears based on classifications and factors.

	Prevalence
	*n* (%)
**Classifications**	
Class	
1	1 (4%)
2	8 (32%)
3	1 (4%)
4	11 (44%)
5	2 (8%)
6	2 (8%)
Stage	
A	1 (4%)
B	5 (20%)
C	11 (44%)
D	8 (32%)
**Gender**	
Male	12 (48%)
Female	13 (52%)
**Tooth type**	
Maxillary	
Central incisors	5 (20%)
Lateral incisors	4 (16%)
Canines	2 (8%)
Premolars	1 (4%)
Molars	3 (12%)
Mandibular	
Central incisors	3 (12%)
Lateral incisors	2 (8%)
Canines	0 (0%)
Premolars	2 (8%)
Molars	3 (12%)
**Stage of assessment**	
Pre‐operative	21 (84%)
Intra‐operative	0 (0%)
Post‐operative	0 (0%)
Review	4 (16%)
**Previous root canal treatment; if no, pulp sensibility tests response**	
No	7 (28%)
Positive	3 (12%)
Negative	2 (8%)
Unspecified	2 (8%)
Previously initiated therapy	2 (8%)
Previously treated	16 (64%)
**Pre‐operative pain**	
No	10 (40%)
Yes	13 (52%)
Unspecified	2 (8%)
**History of dental trauma**	
No	1 (4%)
Yes	6 (24%)
Unspecified	18 (72%)
**Habit of bruxism**	
No	15 (60%)
Yes	6 (24%)
Unspecified	4 (16%)
**Crestal bone loss**	
Mild (≤ 30%)	7 (8%)
Moderate (40%–60%)	2 (8%)
Severe (≥ 70%)	16 (56%)
**Swelling +/ sinus tract**	
No	11 (44%)
Yes	13 (52%)
Unspecified	1 (4%)
**Tooth mobility**	
No	3 (12%)
Grade 1	16 (64%)
Grade 2	1 (4%)
Grade 3	2 (8%)
Unspecified	3 (12%)
**Periapical lesion**	
≤ 10 mm	22 (88%)
> 10 mm	3 (12%)
**Periodontal probing depths**	
≤ 3 mm	8 (32%)
4‐6 mm	2 (8%)
≥ 7 mm	13 (52%)
Unspecified	2 (8%)
**Post**	
Absent	11 (44%)
Present	5 (20%)
Not applicable	9 (36%)
**Restoration**	
Crown (definitive or temporary)	9 (36%)
Composite	7 (28%)
Temporary restoration	2 (8%)
No restoration	7 (28%)
**No. of proximal contacts**	
0	1 (4%)
1	3 (12%)
2	21 (84%)

For cases with cemental tears, the gender distribution was comparable between males and females, and the average age of subjects was 58.7 years old, with males and females being 56.4 and 61 years old, respectively (Tables 3 and 4). Over half of the cases with cemental tear/s were upper and lower incisors, while molars only constituted 1/4 (Tables 3 and 4). Among all CBCT scans taken, merely 15% were done during the review of an endodontically‐treated tooth; all others were taken as part of the initial pre‐operative assessment. Most cases with cemental tear/s (i.e., 72%) were either previously root‐treated or had treatment initiated. The history of dental trauma was frequently unspecified in the clinical record, while bruxism was reported by 6 out of 25 (24%) of the patients. Over half of the cases (i.e., 52%) had pre‐operative pain, severe crestal bone loss of ≥ 70%, complete apico‐marginal bone loss, swelling and/or sinus tract or periodontal probing depth ≥ 7 mm. Grade 1 mobility was present in 16 out of 25 (64%) of the cases, while only 3 out of 25 (12%) of the cases presented with a periapical lesion > 10 mm or had less than two proximal contacts (Table 4).

**FIGURE 3:**
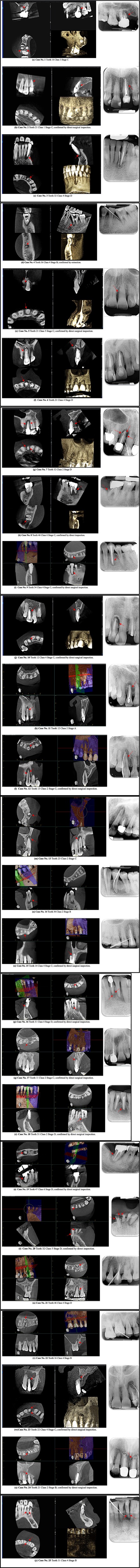
(a–y) Pre‐operative or pre‐surgical CBCT images and periapical radiographs representative of 25 cases with cemental tears. A red arrow indicates cemental tears.

Among the 25 cases diagnosed with cemental tears, 15 cases were identified using both PR and CBCT (Figure 3). The remaining 10 cases were detectable exclusively on CBCT imaging (Figure 3). The overall sensitivity and specificity for intraoral PR, using CBCT as the reference standard, were 0.60 and 1.00, respectively (Table 5).

**TABLE 5:** Diagnostic accuracy of PR in detecting cemental tears, using CBCT as the reference standard.

Imaging tool	Sensitivity	Specificity	PPV	NPV	Reliability
PR	0.6	1.0	1.0	0.98	0.98

## 4 Discussion

The scientific evidence available on the epidemiology of cemental tears remains scarce. Keskin and Güler (2017) reported a prevalence of cemental tears of 0.9%, whereas Özkan and Özkan (2020) reported a slightly higher prevalence of 1.9%. The former study evaluated the presence of cemental tears using two‐planar radiographic imaging alone; this could have underestimated the actual prevalence because, in our research, periapical radiographs alone could only detect 60% of the cases with cemental tears. Özkan and Özkan (2020) study evaluated more than 800 CBCT images taken at a lower resolution and larger FOV than the current study, which might lower the sensitivity of detecting cemental tears. In contrast, our study used a combination of periapical radiographs and high‐resolution, small FOV CBCT scans as the assessment imaging tools. This could explain why the current study found a higher prevalence of 5.6% among the patients referred for endodontic assessment than the two previous studies. Thus, the potential presence of cemental tears should always be considered in the differential diagnosis, and the use of CBCT should be contemplated when cemental tears are suspected. The prevalence findings of this study can be generalisable to the patient population seen and treated primarily through endodontic referrals, making them relevant to various clinical settings. These findings are especially pertinent to endodontic specialist practices, dental clinics or institutions with a primary focus on endodontic care.

To mitigate clustering effects in statistical analysis, the unit of measurement was defined as the ‘patient’ rather than the ‘tooth’ in the study on the prevalence of cemental tears. By including only one tooth per patient, the aim was to account for patient‐specific factors that could introduce confounding variables to the analysis of correlating factors. However, this method may have led to an underestimation of the actual prevalence of cemental tears. When further investigation was conducted, it was revealed that only one of the 25 cases of cemental tears involved multiple teeth, indicating a minimal impact on the overall prevalence reported in the study. Furthermore, the study's scope might have been constrained by the limited field of view (FOV) of the cone‐beam computed tomography (CBCT), which only captured a few neighbouring teeth in relation to the tooth under investigation, potentially overlooking details about other teeth with cemental tears. While this limitation could be mitigated by employing a larger FOV or multiple small FOV CBCT scans of different regions, it is essential to consider the trade‐off between image quality, the accuracy of detecting cemental tears and the ethical considerations involved in obtaining such images. The potential limitations of CBCT scans, such as increased radiation exposure and cost, should always be considered.

Most cases with cemental tears were classified as either Class 2 and 4 or Stage C and D. Class 2 is defined as cemental tears that cannot be clinically probed and are not associated with any deep periodontal pockets; the torn cemental fragments and/or their associated bony lesions involve the root apex without crestal bone loss (Lee et al. 2021) (Figure 2). Meanwhile, Class 4 is defined as cemental tears that cannot be clinically detected and are not associated with deep periodontal pockets; the torn cemental fragments and/or their associated bony lesions show apico‐marginal involvement (Lee et al. 2021) (Figure 2). Stages C and D are defined as cemental tears and the associated bony lesions involving three or all root surfaces, respectively (Lee et al. 2021) (Figure 2). Logically, periapical bone loss, as observed in Class 2 and 4 cases, usually affects three or more apical root surfaces, contributing to the increased prevalence of Stages C and D in this study. These reasons may explain why referring clinicians initially misinterpreted these cases as stemming from endodontic issues, leading to their referral to the Postgraduate Endodontic Clinic.

This study concurred with the findings of the two other prevalence studies that age and gender were not significantly correlated with cemental tears (Keskin and Güler 2017; Özkan and Özkan 2020). Eighteen out of 25 (i.e., 76%) cases with cemental tears were found to have received root canal treatment or had the treatment initiated. As suggested by Lee and co‐workers (2021), this might reflect prior misdiagnosis and mistreatment in the first place, which was eventually judged as failed treatment or post‐treatment disease and hence the referral. Despite this, this study did not find previous root canal treatment significantly correlated with cemental tears (Table 1), which corroborated the findings of Özkan and Özkan (2020).

This study found that cemental tears were significantly more prevalent in upper and lower incisors than in their molar counterparts. There were several plausible reasons: (i) incisors are known to suffer a higher prevalence of dental trauma than molars (Bauss et al. 2004; Kaste et al. 1996); (ii) both the clinicians and patients were more willing to investigate and treat the diseases to retain incisors, whereas molars were more likely to be extracted (Alesia and Khalil 2013; Ng et al. 2010); and (iii) single‐rooted incisors were more susceptible to occlusal trauma due to root convergence, as opposed to multi‐rooted molars with divergent roots that offer stability and the ability to withstand increased occlusal and lateral forces (Anggraini et al. 2018).

Severe crestal bone loss of ≥ 70% was a significant factor associated with cemental tears. This explains why cases with cemental tears could easily be misdiagnosed as primary endodontic or periodontal diseases and/or combined endodontic–periodontal diseases (Lee et al. 2021). As previously mentioned, the increased prevalence of severe crestal bone loss in cases of cemental tears could reflect an increased tendency for mis‐ or delayed diagnosis and subsequent mismanagement, resulting in extensive bone loss by the time referrals were made. Severe crestal bone loss would undoubtedly affect the treatment prognosis, resulting in lowered survival and loss of many teeth that could have been salvaged if diagnosed and treated earlier. Besides, increased tooth mobility and secondary occlusal trauma are often the side effects of reduced periodontal support (Harrel 2003). Establishing the cause‐and‐effect relationship between crestal bone loss and the development or propagation of cemental tears presents challenges. It remains unclear whether reduced crestal bone support predisposes a tooth to cemental tears or if the destructive consequences of cemental tears lead to increased crestal bone loss. Both factors likely contribute to varying extents in the pathogenesis of cemental tears.

This study also identified that increased tooth mobility was significantly correlated with cemental tears. Primary and secondary occlusal trauma are the common causes of increased tooth mobility (Fan and Caton 2018; Lee et al. 2021), which might be attributed to occlusal interference, parafunctional habit, heavy occlusal loading, unfavourable root morphology and/or reduced periodontal support (Branschofsky et al. 2011; Fan and Caton 2018). The authors postulated that occlusal trauma could act as the primary causative factor in the development and propagation of cemental tears, or it could occur secondarily due to reduced periodontal support and increased bone loss in teeth suffering from cemental tears. The current study demonstrates that periodontitis is frequently implicated in teeth suffering from cemental tears, whose prognosis could be negatively affected by occlusal trauma and increased mobility, or vice versa (Harrel 2003). This reflects the close‐knit interrelationship between these factors.

Interestingly, root‐filling with adequate quality was also a significant factor. This might be the biological cost of achieving homogenous and adequately extended obturation, as the obturation instruments and techniques could induce stresses on the root dentine (Saw and Messer 1995). For example, heat is often required to thermoplasticise the gutta‐percha; improper control of the temperature rise could cause excessive desiccation, cemental structural damage and/or periodontal tissue injury, resulting in crack initiation and/or propagation on the root surface (Donnermeyer et al. 2018; Viapiana et al. 2014). Furthermore, the mechanical compaction force arising from the finger spreader in the lateral compaction technique or binding of the dentinal canal luminal wall from endodontic pluggers in warm vertical or continuous wave compaction techniques could exert excessive strain on the root dentine (Blum et al. 1997). This is an area worthy of further research.

Several limitations identified in this study are also intrinsic to all retrospective observational studies. It was acknowledged that excluding cases due to inadequate clinical documentation and records may have resulted in overlooking cases where cemental tears were present, potentially leading to an underestimation of prevalence. This may be alleviated with a well‐designed prospective study. Occlusal interference was not studied because it was rarely documented in the patient's clinical record, and hence, insufficient data relating to this factor could be gathered. The authors acknowledged that occlusal interference is a critical factor that warrants further investigation due to its potentially detrimental effects on root structures during function or parafunction, contributing to the prevalence of cemental tears. The patient's dental records also often contained incomplete or unspecified information regarding various factors that could potentially introduce bias. Some factors, such as a history of dental trauma, bruxism, hard diet and mobility, consisted of at least 10% of unspecified data (Table 1). These factors are generally considered to be associated with heavy occlusal loading, occlusal trauma and traumatic impacts, which can impose increased stress on the root structure, potentially contributing to cemental tears (Jeng et al. 2018; Lee et al. 2021). In particular, the history of dental trauma exhibited as much as 83.1% missing information with borderline significance of *p* = 0.073, which raises concerns about the reliability of the result. There was a possibility of a Type II statistical error—meaning a factor might actually have a significant correlation with cemental tears but remained undetected due to incomplete data. This underlines the importance of comprehensive data collection and analysis, as missing information can obscure true relationships. Additionally, these unaccounted factors could act as confounders, potentially influencing the interpretation of other significant variables identified in the study. Recognising and addressing these limitations is essential for a more accurate understanding of the factors contributing to cemental tears and for guiding future research directions.

Direct inspection and/or histopathological confirmation remained the gold standard for definitive diagnosis of cemental tears (Lee et al. 2021). Although 15 out of the 25 cases (60%) with cemental tears were confirmed through direct inspection (i.e., 2 cases via non‐surgical means and 13 cases via surgical means), it was still a limitation of this study that not all cases could be confirmed through these approaches (Figure 3). An invaluable comparison would involve assessing the diagnostic accuracy of intraoral PR and cone‐beam computed tomography (CBCT) in detecting cemental tears, with histopathologic findings and direct inspection serving as the gold standard. Our research team is currently undertaking a study to investigate this aspect. The authors recognised that relying on a single observer may lead to bias. Ideally, multiple assessors should have been recruited. However, sourcing experienced assessors proficient in diagnosing cemental tears was challenging due to the rarity of this condition. To address this issue, our research team is undertaking a study that involves multiple observers in diagnosing cemental tears, aiming to investigate potential differences in diagnostic accuracy among assessors with varied clinical training backgrounds and experience levels. Even though the intra‐observer reliability was excellent in this study, all the cases with cemental tears detected are illustrated in Figure 3a–y to improve the credibility of the current findings.

## 5 Conclusion

The prevalence of cemental tears among patients referred for endodontic evaluation was notably high at 5.6%, underscoring the importance of incorporating this pathology into differential diagnostic considerations within endodontic practice. An increased proportion of cases exhibited periapical bone loss, which presents a substantial risk for misdiagnosis and inappropriate management if lesions are solely attributed to endodontic origins. Clinicians should exercise vigilance regarding increased susceptibility to cemental tears in incisors, teeth exhibiting mobility, those with pronounced crestal bone loss, and teeth with adequately filled root canals, as many of these features are also characteristic of periodontal–endodontic lesions. The use of cone‐beam computed tomography (CBCT) is crucial for accurate diagnosis, facilitating effective treatment planning and improving clinical outcomes in endodontic care.

## Author Contributions

Conceptualisation: A.H.C. Lee, C.F. Zhang, M.C.M. Wong. Data curation: A.H.C. Lee, A.W.K. Yeung. Formal analysis: A.H.C. Lee, M.C.M. Wong. Funding acquisition: C.F. Zhang. Investigation: A.H.C. Lee. Methodology: A.H.C. Lee, C.F. Zhang, M.C.M. Wong. Project administration: A.H.C. Lee. Resources: A.W.K. Yeung. Software: A.W.K. Yeung. Supervision: C.F. Zhang, M.C.M. Wong. Validation: A.H.C. Lee, M.C.M. Wong, A. Sigurdsson. Visualisation: A.H.C. Lee, C.F. Zhang. Writing – original draft preparation: A.H.C. Lee, C.F. Zhang, M.C.M. Wong, A. Sigurdsson. Writing – review and editing: A.H.C. Lee, C.F. Zhang, M.C.M. Wong, A. Sigurdsson.

## Conflicts of Interest

The authors declare no conflicts of interest.

## Supporting information


**Data S1:** iej70042‐sup‐0001‐DataS1.docx.

## Data Availability

The data that support the findings of this study are available from the corresponding author upon reasonable request.
