# Supplementary material for: Prevalence, Classification and Factors Associated With Cemental Tears—A Retrospective Observational Cross‐Sectional Study in a Postgraduate Endodontic Clinic
Source: Int Endod J. 2025 Sep 30;59(1):57–79. doi: 10.1111/iej.70042 (PMC12701744; doi:10.1111/iej.70042)
Supplement: Supplementary file 1 — Data S1: iej70042‐sup‐0001‐DataS1.docx. [file IEJ-59-57-s001.docx]

**PROBE 2023**

**Checklist of items to be included when reporting observational studies in Endodontics***

| Section/  Topic | Item Number | Checklist items | **Reported on page number** |
| --- | --- | --- | --- |
| Title | 1a | The specific area(s) of interest must be provided using words and phrases that identify the clinical problem(s) and focus of the study | 1 |
|  | 1b | The study design must be included in the Title, e.g., cross-sectional, cohort, case-control, case-series etc. | 1 |
| Keywords | 2a | Keywords indicating the specific area(s) of interest using MeSH terms or other more applicable terms must be included | Title page |
| Abstract | 3a | The Introduction/Background must briefly explain the rationale or justification for the study | 1 |
|  | 3b | The aim(s)/objective(s) of the study must be provided | 1 |
|  | 3c | The Methodology must provide (where relevant) essential information on the nature of the study design (retrospective, cross-sectional, prospective, etc.), setting, location(s), and relevant dates, including periods of recruitment, exposure, follow-up, outcome(s) assessed and statistical analysis | 1 |
|  | 3d | The Results must describe the number of subjects that were included and analysed as well as the most significant results for all experimental and control groups. The results of statistical analysis must be reported in terms of unadjusted and confounder-adjusted outcomes (if relevant). Adverse events or side-effects must also be reported if present or confirmed as absent | 1 |
|  | 3e | The Conclusion must interpret and summarise the primary aim/objective and main findings as well as emphasise the clinical implications | 2 |
|  | 3f | The source(s) of funding must be provided | 34 |
| Introduction | 4a | The clinical problem/question, scientific background and rationale for the study must be provided, including the gap(s) or inconsistencies in the existing knowledge base | 3-4 |
|  | 4b | The primary and, if applicable, any additional/secondary aim(s) and objective(s) of the study must be provided, including any pre-specified hypotheses | 4 |
| Methods  *Ethics* | 5a | The details (name, reference number, date) of the approval or exemption granted by an ethics committee, such as an Institutional Review Board, must be provided | 5 |
|  | 5b | The process used for obtaining and storing informed consent must be provided | 5 |
| *Study design* | 5c | The key elements of the study design must be described early in the Methods section | 5 |
| *Setting* | 5d | The details of setting(s), location(s), socioeconomic status of participants (if available) and relevant dates, including periods of recruitment, exposure, follow-up, and data collection must be provided | 5-6 |
| *Sample size* | 5e | Information on how the sample size was determined *a priori* must be provided as well as the rationale for sample size calculation, preferably with reference to the published literature or a pilot study with additional detail as to why the defined sample size makes the study worthwhile | 5 |
| *Participants – unmatched studies* | 5f | All studies should include inclusion/exclusion criteria as well as the sources and methods of participant selection. Methods of follow-up must also be provided in cohort studies and the rationale for the choice of ‘cases’ and ‘controls’ in case-control studies | 5-6 |
| *Participants – matched studies* | 5g | For matched studies (e.g., cohort, case-control) the matching criteria and the numbers of participants in each group must be provided | Not applicable |
| *Variables* | 5h | All outcomes, exposures, predictors, potential confounders, and effect modifiers must be defined clearly | 7-8 |
| *Data sources/ measurement* | 5i | Sources of data and details of the methods of assessment (measurement) for each variable of interest must be provided | 6-7 |
| *Bias* | 5j | Efforts taken to identify and address potential sources of bias muste provided | 7 |
| *Quantitative variables* | 5k | The handling of quantitative variables in the analyses must be explained. Decisions on how groupings were made and/or how category boundaries were defined for continuous variables must be described | 9,  14-20 |
| *Statistical methods* | 5l | All statistical methods, including those used to control of confounding factors in the study and in the analysis of the data, must be described | 8-9 |
|  | 5m | The methods used to examine subgroups and interactions must be described, if applicable | 8-9 |
|  | 5n | Missing data (e.g. drop-outs, data not reported) must be addressed and described | 8-9 |
|  | 5o | The analytical methods that take account of the sampling strategy (if applicable) in *Cross-sectional studies* must be described | 8 |
|  | 5p | Sensitivity analyses, must be described when used | Not applicable |
| Results *Participants* | 6a | The number of participants in each stage of the study (i.e., eligibility, recruitment, available at follow-up and included in analyses for relevant outcome(s)) must be described | 9 |
|  | 6b | Reasons for non-participation (e.g., not eligible, losses/drop-outs) must be described | Not applicable |
| *Dates* | 6c | Changes in baseline dates of recruitment, follow-up, and study duration reported in the Methodology must be described, if applicable | Not applicable |
| *Descriptive data* | 6d | The baseline demographic and clinical characteristics of study participants as well as information on exposures and potential confounders must be provided | 9-11,  12-20 |
|  | 6e | The number of participants with missing data must be provided for each variable. If relevant, follow-up times should be summarised clearly and accurately (e.g., average or total time) | 14-20 |
| *Outcome data* | 6f | Information on number of outcomes or summary measures over time must be described | 9-11,  12-20 |
|  | 6g | For multivariable analyses developing risk profiles or reducing the effect of confounders, the effect of all included independent variables may be reported, as well as their effects on the prediction model (if applicable) | 10,  16-20 |
| *Main results* | 6h | Unadjusted (or uncorrected or crude) estimates and, if applicable, confounder-adjusted estimates and their precision (e.g., 95% confidence intervals) must be described. Which confounders were adjusted for and why they were included must also be described | 20 |
|  | 6i | Results in terms of relative risk should also be translated to absolute risk for a meaningful time period, if relevant | Not applicable |
| *Additional analyses* | 6j | The results from any other analyses (e.g., sensitivity, subgroup analyses) must be described, if applicable, as well as adjusted analyses, distinguishing pre-specified from exploratory | Not applicable |
| Discussion  *Key results* | 7a | The main findings must be summarized with reference to the study aim(s)/objective(s) | 30-34 |
| *Rationale* | 7b | The rationale for inclusion/exclusion criteria, exposure, and duration must be provided | 30 |
| *Clinical relevance* | 7c | An explanation of the clinical relevance of the primary and any additional/secondary outcome(s) must be provided | 30-34 |
| *Strength* | 7d | The strength(s) of the study must be provided | 30 |
| *Limitations* | 7e | The limitations of the study must be provided - addressing the sources of potential bias, imprecision, study design, study size and potentially important but missing confounding variables. Both direction and magnitude of any potential bias must be discussed | 33-34 |
| *Summary and validity* | 7f | The discussion of the strength and limitations should be summarized in an overall assessment of the internal validity of the study | 33-34 |
| *Interpretation* | 7g | A detailed interpretation consistent with results, balancing benefits and harms, and considering other relevant evidence must be provided | 30-33 |
| *Generalisability* | 7h | The generalizability (external validity, applicability, real-world relevance etc.) of the study findings must be discussed | 30-32 |
| *Future directions* | 7i | Implication for future research and clinical practice must be described | 33-34 |
| Conclusion(s) | 8a | Explicit conclusion(s) from the study must be provided and address all the aims/objectives | 34 |
| Funding details | 9a | All sources of funding and other support (such as supply of drugs, equipment etc.) as well as the role of funders must be acknowledged and described | 34 |
| Conflict of interest | 10a | An explicit statement on conflicts of interest must be provided, together with full affiliations of every author(s) | 34 |
| Quality of images (if applicable) | 11a | Details of the equipment, software and settings used to acquire the image(s) must be described in the text or legend (if applicable) | 6-7 |
|  | 11b | The reason why the image(s) was acquired and the rationale for its inclusion in the manuscript must be provided in the manuscript. A justification for all images that involve ionising radiation must be included | 7-8  21-29  33 |
|  | 11c | The circumstances (conditions) under which the image(s) were viewed and evaluated by the author(s) must be provided in the text | 7 |
|  | 11d | The resolution, any magnification of the image(s) or modifications/enhancements (e.g., adjustments for brightness, colour balance, magnification, image smoothing, staining, etc.) that were carried out must be described in the text or figure legend | 6-7 |
|  | 11e | Patient(s) identifiers (names, patient numbers) must be removed for General Data Protection Regulation (GDPR) and to ensure they are anonymized or de-identified in all images | 12-13  21-29 |
|  | 11f | An interpretation of the findings (meaning and implications) from the image(s) must be provided in the text | 9-11 |
|  | 11g | The figure legend associated with each image must describe clearly what the subject is and what specific feature(s) is illustrated. If cases are offered to illustrate descriptions of a cohort, then the age, gender, ethnicity, and other specific attributes that are relevant to the cohort should be provided | 21-29 |
|  | 11h | Markers/labels must be used to identify the key information in the image(s) and defined in the figure legend | 21-29 |
|  | 11i | The figure legend of each image must include an explanation on whether it is pre-, intra- or post-treatment and follow-up and, if relevant, how images were standardised over time | 29 |

*** Nagendrababu V, Duncan HF, Fouad AF, Kirkevang LL, Parashos P, Pigg M, Vaeth M, Jayaraman J, Suresh N, Arias A, Wigsten E, Dummer PMH. PROBE 2023 guidelines for reporting observational studies in Endodontics: A consensus-based development study. Int Endod J. 2022 Nov 23. doi: 10.1111/iej.13873.**
